# Transcriptional Responses of Chilean Quinoa (*Chenopodium quinoa* Willd.) Under Water Deficit Conditions Uncovers ABA-Independent Expression Patterns

**DOI:** 10.3389/fpls.2017.00216

**Published:** 2017-03-08

**Authors:** Andrea Morales, Andres Zurita-Silva, Jonathan Maldonado, Herman Silva

**Affiliations:** ^1^Centro de Estudios Avanzados en Zonas Áridas, Universidad de La SerenaLa Serena, Chile; ^2^Instituto de Investigaciones Agropecuarias, Centro de Investigación IntihuasiLa Serena, Chile; ^3^Laboratorio de Genómica Funcional & Bioinformática, Departamento de Producción Agrícola, Facultad de Ciencias Agronómicas, Universidad de ChileSantiago, Chile

**Keywords:** drought tolerance, Andean grain, Salares and coastal/lowlands genotypes, RNA-Seq, qPCR

## Abstract

**HIGHLIGHTS**

- R49 genotype displayed best performance on selected physiological parameters and highest tolerance to drought.
- R49 drought over-represented transcripts has exhibited 19% of genes (306 contigs) that presented no homology to published databases.
- Expression pattern for canonical responses to drought such as ABA biosynthesis and other genes induced in response to drought were assessed by qPCR.

Global freshwater shortage is one of the biggest challenges of our time, often associated to misuse, increased consumption demands and the effects of climate change, paralleled with the desertification of vast areas. *Chenopodium quinoa* (Willd.) represents a very promising species, due to both nutritional content and cultivation under water constraint. We characterized drought tolerance of three Chilean genotypes and selected Genotype R49 (Salares ecotype) based upon Relative Water Content (RWC), Electrolyte Leakage (EL) and maximum efficiency of photosystem II (F_v_/F_m_) after drought treatment, when compared to another two genotypes. Exploratory RNA-Seq of R49 was generated by Illumina paired-ends method comparing drought and control irrigation conditions. We obtained 104.8 million reads, with 54 million reads for control condition and 51 million reads for drought condition. Reads were assembled in 150,952 contigs, were 31,523 contigs have a reading frame of at least 300 nucleotides (100 aminoacids). BLAST2GO annotation showed a 15% of genes without homology to NCBI proteins, but increased to 19% (306 contigs) when focused into drought-induced genes. Expression pattern for canonical drought responses such as ABA biosynthesis and other genes induced were assessed by qPCR, suggesting novelty of R49 drought responses.

## Introduction

Plants are sessile organisms that need to respond to a wide array of abiotic and biotic stresses. This condition confers strong selective pressures on their local adaptation to different environments. The selective pressure at the abiotic level implies stress responses, that includes numerous and complex environmental conditions, such as light intensity, temperature, salinity and drought. Therefore, understanding abiotic stress responses at the physiological and genomic level is a relevant issue to provide an essential foundation for future breeding and genetic engineering efforts. Indeed, roughly 75% of the world's freshwater supplies are utilized in agriculture, and the increasing climatic variability and the demographic pressures have led to ecosystem degradation and have exacerbated the vulnerability to drought and other abiotic stress factors (Food Agriculture Organization of the United Nations, 2011). Consequently, increasing crop productivity under conditions of limiting water availability is of major importance (Avramova et al., 2015). Research on drought responses in Arabidopsis, maize, tomato and rice among others plants, have determined that a large number of genes as well as signal transduction pathways are involved in drought responses (Shinozaki et al., 2003; Shinozaki and Yamaguchi-Shinozaki, 2007). The identification of genes regulated by drought conditions has a high significance, because it provides a comprehensive understanding of the transcriptional responses and the identification of stress responsive promoters and cis-elements.

Quinoa (*Chenopodium quinoa* Willd.) is an Andean native crop that belongs to the Amaranthaceae family. It is an allotetraploid plant (2*n* = 4× = 36) with a genome size estimated of 967 Mbp (Mujica and Jacobsen, 2006; Stevens et al., 2006) and sequenced very recently with a total assembly size of 1.39 gigabases (Gb) (Jarvis et al., 2017), which shows disomic inheritance for most qualitative traits (Simmons, 1971; Risi and Galwey, 1984; Ward, 2000; Maughan et al., 2004). Quinoa was domesticated and has been cultivated in the Andes for the last 7,000 years before present (BP). It's diversity is comprised by five major ecotypes related to different sub-center origins that include: Highlands (Peru and Bolivia), Inter-Andean valleys (Bolivia, Colombia, Ecuador and Peru), Salares (Bolivia, Chile and Argentina), Yungas (Bolivia) and Lowlands (Chile) (Risi and Galwey, 1989a,b; Bertero et al., 2004; Zurita-Silva et al., 2014; Bazile et al., 2015). Among nutritional characteristics it is know that quinoa's seeds have an exceptional balance between oil (4–9%), protein (averaging 16%, with high nutritional relevance due to optimal balance of essential amino acid content) and carbohydrates (64%) (Bhargava et al., 2006; Vega-Gálvez et al., 2010). Moreover, its high starch content (51–61%) enabling flour production (Mastebroek et al., 2000; Repo-Carrasco et al., 2003; Stikic et al., 2012) but with the advantages of gluten absence, which has allowed the development of foods for consumers with celiac disease (i.e., people allergic to gluten) (Jacobsen, 2003). Additionally, quinoa is a good source of vitamins, oil with high linoleate and linolenate content (55–66% of the lipid fraction), natural antioxidants such as α- and γ-tocopherol, and a wide range of minerals (Repo-Carrasco et al., 2003; Vega-Gálvez et al., 2010; Fuentes and Bhargava, 2011; Stikic et al., 2012; Ruiz et al., 2014). Interestingly, quinoa consumption may lead to comparatively lower weight gain, and improved lipid profile and potential antioxidant effects, physiological outcomes that have been linked to bioactive compounds, such as saponins, quinoa proteins, polyphenolic compounds and 20-hydroxyecdysone by yet unknown mechanisms (Simnadis et al., 2015). Considering the attributes and potential to contribute to food security worldwide, the draft genome sequence of an inbred line has been recently published, comprising a free-access Quinoa Genome DataBase (QGDB), which will provide insights into the mechanisms underlying agronomically important traits of quinoa (Yasui et al., 2016).

Quinoa is an interesting abiotic stress tolerant crop that should be adopted as a model because has a good tolerance to high salinity, boron, light intensity and drought (Orsini et al., 2011; Ruiz-Carrasco et al., 2011; Zurita-Silva et al., 2014; Razzaghi et al., 2015; Ruiz et al., 2016). In particular quinoa has a good tolerance to water shortage that is due to its intrinsic lower water requirement, the capability to regain its original level of photosynthesis after a drought period, and both slow growth and smaller leaf area during acclimation (Galwey, 1989; Jensen et al., 2000; Jacobsen et al., 2003, 2009; Sun et al., 2014). There are others factors than can be considered for this tolerance i.e., high instantaneous photosynthetic efficiency in drought conditions (Winkel et al., 2002; Bosque Sanchez et al., 2003); leaf shedding (Jensen et al., 2000); its higher root branching and foraging capacity of root system (Alvarez-Flores et al., 2014), and the presence of leaf vesicles containing calcium oxalate, which could reduce transpiration (Jensen et al., 2000; Siener et al., 2006). It's noteworthy that this tolerance to water deficit has allowed reaching harvests with only 75 mm of rainfall (Martínez et al., 2009). In this work we characterized three Chilean genotypes (Martínez et al., 2015), one corresponding to Salares and two from Lowlands native ecotypes, which frequently experience water deficit during growth season, in terms of drought tolerance. Later, for the most tolerant genotype, a RNA-seq analysis of the transcriptome was performed to explore candidate transcripts under contrasting water conditions i.e., deficit and normal irrigation. A deep bioinformatics analysis is presented that assessed a putative signal transduction pathway involved in quinoa responses to drought. To validate the assembly of the contigs, primer pairs were designed to amplify the sequences of 15 selected transcripts, related to drought response and ABA biosynthesis and to study the expression level of target genes through Real Time -quantitative PCR (RT-qPCR).

## Materials and methods

### Plant material

Three Chilean genotypes of *Chenopodium quinoa* Willd. were used in this study: R49, from Colchane, Tarapacá Region, 3,850 m altitude (Salares ecotype); PRJ, from Cahuil, O'Higgins Region, 39 m altitude and BO78 from Collipulli, Araucanía Region, 243 m altitude (the last two coastal/lowland ecotypes; Table 1). The seeds were kindly provided by the National Seed Bank of Chile managed by Instituto de Investigaciones Agropecuarias INIA Intihuasi (Vicuña, Chile).

**Table 1:** **Chilean genotypes studied: ecotype and geographical origin**.

**Genotype**	**Ecotype**	**Provenance**	**Latitude/ Longitude**	**Altitude (m.a.s.l.)**	**Rainfall (a.a. mm)**
R49	*Salares*	Colchane county	19°25′; 68°35′	3,850	187
PRJ	Coastal /lowlands	Cahuil	34°29′; 72°00′	39	641
BO78	Coastal /lowlands	Collipulli	37°57′; 72°26′	243	1,324

*m.a.s.l., meters above sea level; a.a., annual average millimeters*.

### Plant growth and stress conditions

Experiment consisted of 12 pots each containing two plants for each genotype that were grown in 1L pots with a soil-sand mixture (1:1) with irrigation every 2 days and temperatures that fluctuated between 12 and 32°C during 4 weeks at greenhouse conditions. To select the genotypes tolerant to drought, at the fourth week since germination half of the plants from each genotype were maintained as control in the same irrigation regimen, and the other half were deprived of water, and distributed in six blocks that were set up according to a randomized block design. The experiments were performed in La Serena, Coquimbo Region, Chile (29°54′; 71°15′) during summer (January).

To determinate the most drought tolerant genotype, we analyzed the following physiological parameters: (1) Relative Water Content (RWC) as described previously by Barrs and Weatherley (1962), (2) Electrolyte Leakage (EL) as described by Pinhero et al. (1997), and F_v_/F_m_ determination (Woo et al., 2008). The experiment was done using 1-month-old plants under a water shortage condition (dry season). The starting of the experiment was ceasing the plants irrigation and the measurement of the physiological parameters previously mentioned. For F_v_/F_m_ determination, three different leaves by treatment were measured from dark-adapted plants during 30 min, and then were subjected to an initial saturating pulse of 900 μmol photons m^−2^ s^−1^, followed by a 40″ delay in darkness and subsequently 10' of actinic illumination with saturating flashes at 20″ intervals, using a foliar chamber 6400-40 coupled to LI-6400 fluorometer (LICOR), and were calculated following reported methods (Woo et al., 2008).

For the transcriptome approach, a second experiment was performed using the most drought tolerant genotype. The growth conditions were similar to the previous experiment and plant tissues were collected at the following stages: (a) Stage 1 (D1), where relative water content (RWC) was near 80% (similar to control) and soil potential was significant different between control and drought (−0.6 MPa and −1.3 MPa, respectively); (b) Stage 2 (D2), where leaf RWC was near 50–60% and the soil potential was −1.3 MPa; and (c) Stage 3 (D3), where leaf RWC was near 30% and soil potential was −2.7 MPa. For the control samples, the same stages were used (C1, C2, C3), in which the analyzed parameters were similar to stage 1 control (80% RWC and −0.6 MPa soil potential) (Table S1). The collected tissues (whole plants) were frozen in liquid nitrogen and then placed at −80°C until further use.

### RNA extraction

Total RNA was obtained from plant tissues using the Trizol reagent (Invitrogen Corp., Carlsbad, CA, USA) following the manufacturer's protocol. We extracted the RNA individually from three different plants for each stage (D1, D2, and D3; C1, C2, and C3), and tissues were separated between root and shoot. After quality analysis (Meisel et al., 2005) we composed two different pools (drought and control) using the same quantity from each plant tissue (root and shoot, 1.6 μg each) and sent to Macrogen (http://www.macrogen.com) for Illumina 100 bp paired-ends sequencing procedure.

### Library construction, deep sequencing and *de novo* transcriptome assembly

Library construction and deep sequencing for each sample were performed at Macrogen (Inc. Seoul, South Korea) using Solexa HiSeq2000 platform with the previous construction of a Truseq mRNA library for paired end application according to Macrogen's protocol with an insertion length of 550 bp. The sequence reads were quality trimmed and assembled using the CLC Genome Workbench version 4.8 (CLC Bio: CLC genomics workbench [http://www.clcbio.com]). Trimming was done following parameters: Q ≥ 20; no more than 2 ambiguities; final read length ≥ 50 bp. Reads assembly was done from a pooling of all the paired end short-read data (hybrid assembly) using the following parameters: similarity = 0.95; length fraction = 0.7; insertion/deletion cost = 3; mismatch cost = 3; automatic bubble and word size; minimum contig length of 200 bp to avoid singlets. Paired end range distance was 217 to 442 bp for the control sample and 211 to 380 bp for the drought treatment sample. To compare contigs assembly we used both CLC and Oases 0.1.22 (Schulz et al., 2012) [http://www.ebi.ac.uk/~zerbino/oases]. Contigs were scanned for full length coding sequence (full length CDS) using the tool GETORF from EMBOSS version 6.3.1 (Rice et al., 2000), looking for open reading frame regions between START and STOP codons and a minimum of 300 nucleotides. Considering that drought related proteins described in other models have more than 100 aa, and incomplete genes can't be resolved without a deep genome exploration that is out of the scope of this work, we chose this cutoff to avoid the presence of incomplete genes. Results were filtered through Perl script to obtain the longest predicted CDS by contig.

### Exploratory data analysis

Contigs with predicted full length CDS obtained from the *de novo* hybrid assembly were used as a reference set of transcripts for RNA-seq analysis. Short-read sequence data from control and drought samples were separately mapped against the reference set of assembled transcripts using the CLC Genome Workbench RNA-seq function using the following parameters: similarity = 0.95; length fraction = 0.7; maximum mismatches = 2; unspecific match limit = 10. Paired reads were counted as two, and paired end distances were set as described previously for the assembly.

For exploratory expression analysis, we selected contigs with at least 5 total reads mapped to each hybrid contig to increase the results confidence (Tittarelli et al., 2009). The relative “over/under-represented” gene levels were defined as the number of reads mapped uniquely to the gene. The representation levels were compared using a *Z*-Test (Kal et al., 1999) with the control sample as reference. This test compares counts by considering the proportions that make up the total sum of counts in each sample, correcting the data for sample size. For visual inspection, original over/under-represented values were transformed by Log_10_ method and then normalized by the Quantile method that was the best to fits the results (Bolstad et al., 2003).

### Functional annotation

Functional annotation was performed on assembled contigs with predicted full length CDS by BLAST2GO software (Conesa and Gotz, 2008). We used as input BLASTX results of our contigs against NCBI RefSeqProt database (Pruitt et al., 2007; Mestanza et al., 2015) and Arabidopsis TAIR10 database (The Arabidopsis Information Resource, Genome Release 10 [http://www.arabidopsis.org]) with an e-value cut-off of 1e^−6^. Also, it was performed an INTERPROSCAN analysis (Hunter et al., 2009) with BLAST2GO default parameters. We used BLAST2GO program defaults in all mapping and annotation steps and the False Discovery Rate (FDR) cut-off was set to 0.05% probability level. The data from INTERPROSCAN terms, enzyme classification codes (EC), and metabolic pathways (KEGG, Kyoto Encyclopedia of Genes and Genomes) were merged with GO terms for a wide functional range cover in annotation (Conesa and Gotz, 2008).

### Quantitative real-time PCR analysis

For each sample, 10 μg total RNA was treated with DNAse RNAse-free (Fermentas), 5 μg of which was reverse transcribed in a 20 μL volume using Affinity Script Multiple Temperature cDNA Synthesis Kit (Agilent) primed with oligo dT. The resulting cDNA was diluted to 200 μL with distillate water. Gene-specific primers were designed to span the selected genes using Primer3 software (http://frodo.wi.mit.edu/primer3/). qPCR was carried out on 1 μL diluted cDNA by triplicate using the MaxPro3000P Stratagene Sequence Detection System, Brilliant III Ultra Fast SYBR Green QPCR master mix (Agilent) and primers at a final concentration between 250 and 450 nM. The primers list is shown in (Table S2). *qPCR* analysis was performed and data (±S.D.) represents three biological replicates following previous reports (Ruiz-Carrasco et al., 2011; Mestanza et al., 2015).

### Statistical analysis

A two-way analysis of variance (ANOVA), followed by Tukey's *post-test*, was performed to evaluate the drought effects on relative water content, electrolyte leakage and F_v_/F_m_ determination to compare genotypes among each other, after testing for normality and homogeneity of variances using the Shapiro-Wilkes test and variation coefficient, respectively.

## Results

### R49 exhibits the most drought tolerant phenotype

Based on the altitudinal and latitudinal distribution of quinoa in Chile we analyzed three genotypes representing the biogeographical areas where this staple crop is actually grown. The genotype R49 is representative of the Chilean highlands (Salares ecotype), where 200 mm/year of rainfall are concentrated in only 1 month during the summer rainy season at an altitude of 3,800 m. The PRJ genotype is grown in central coastal Chile with a rainfall of 600 mm/year that is also concentrated during the fall season, and the genotype BO78 is from the southern Chile were the rainfall reaches 1,300 mm and the rain period occur throughout the whole year, the last two genotypes corresponding to coastal/lowland ecotype.

**Figure 1:**
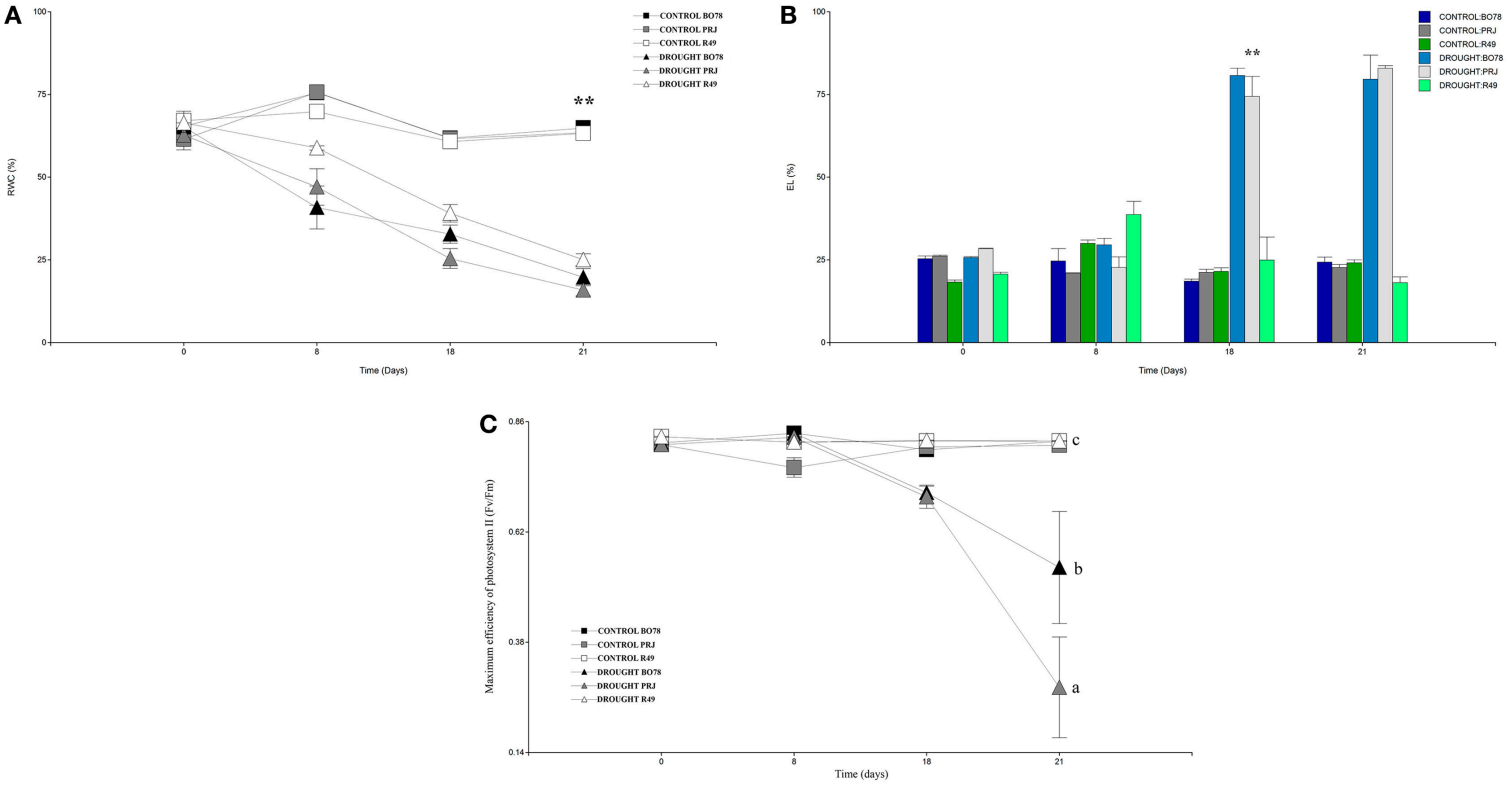
**Physiological performance of quinoa genotypes in days under drought conditions. (A)** Percentage of Relative water content (RWC). **(B)** Percentage of Electrolytic leakage (EL). **(C)** Maximum efficiency of photosystem II (F_v_/F_m_). X-axis represents days under drought. RWC was measured based in the plant total weight that corresponded to water; EL represents the percentage of ions that were released compared to total amount present in the plant; F_v_/F_m_ represents the capacity for photon energy absorbed by photosystem II (PSII) to be utilized in photochemistry under dark- and light-adapted conditions. Values and Bars represent averages and standard deviation (*n* = 3); ^**^ and different letters denote significant differences (*p* < 0.01 and *p* < 0.05 respectively).

Figure 1 shows the genotype responses for relative water content, electrolytic leakage and maximum efficiency of photosystem II (F_v_/F_m_) parameter. The analysis of the relative water content (Figure 1A) showed a gradual decrease for all genotypes from 8 days from the treatment onward thus decreasing their RWC below 30%; the genotype R49 showed a slight difference compared to the others genotypes after 21 days of drought. We could observe a significant difference [*F*_(1.63)_ = 129.01; *p* < 0.0001] since 18 days post treatment compared to PRJ. On the other hand, BO78 showed an intermediate behavior between both genotypes but not significantly different. When measuring electrolytic leakage (Figure 1B), a significant increase [*F*_(1.63)_ = 34.37; *p* < 0.0001] for the central and south genotypes was observed at 18 days, increasing over 70% EL; nevertheless, the Salares genotype did not display major differences through the whole experiment similar to control of all genotypes. The parameter of maximum efficiency of photosystem II (F_v_/F_m_; Figure 1C) significantly decreased [*F*_(1.63)_ = 6.47; *p* < 0.0001] as response to drought treatment for genotypes PRJ and BO78 only from 18 days of drought treatment, in contrast to a stable F_v_/F_m_ ratio in genotype R49 throughout the whole experiment, showing that photosynthetic machinery in R49 leaves remained functional despite prolonged water deficit condition.

### RNA-sequencing, reference transcriptome assembly and gene space of quinoa under drought conditions

Two different samples were sequenced to obtain the R49 quinoa transcriptome. One of the samples corresponded to plants with the drought stress treatment and the other one to control plants as described in materials and methods. After library construction, Illumina paired -end sequencing was performed. 54 million and 51 million reads were obtained from the control and drought conditions respectively. Both reads from control and drought conditions were used to build a reference transcriptome due to the lack of an available quinoa genome at that stage.

**Figure 2:**
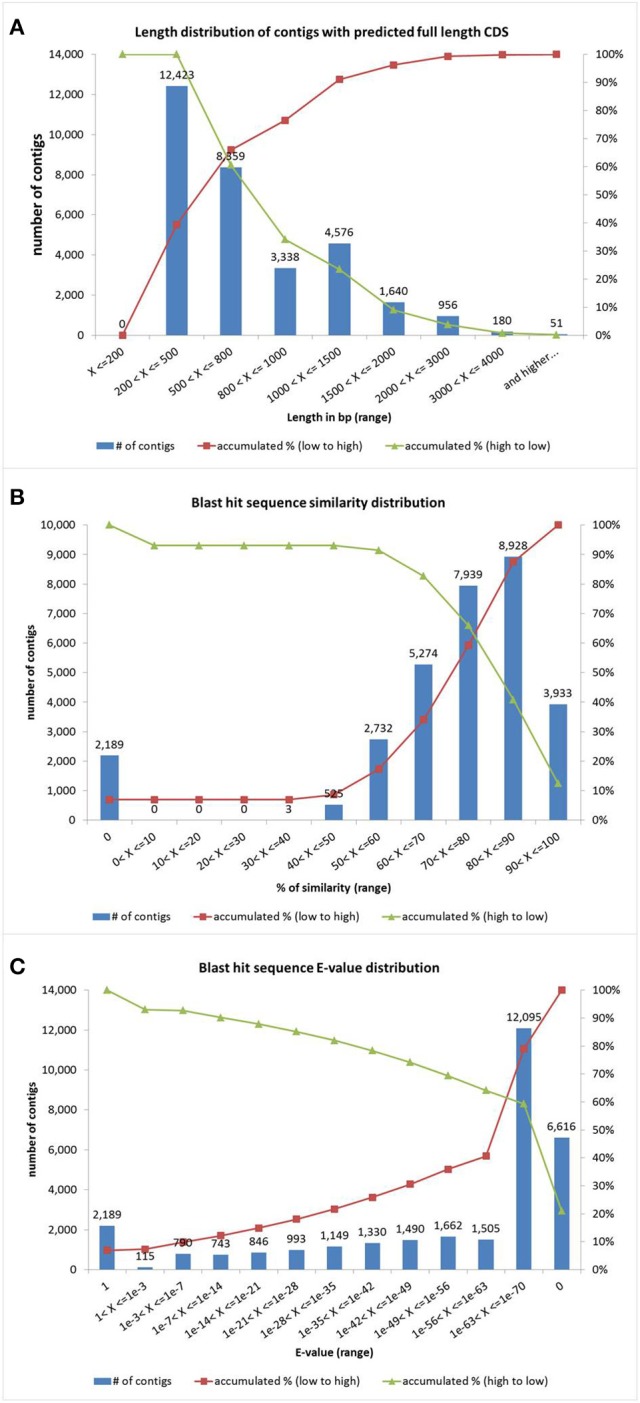
**Identity analysis of quinoa genes. (A)** Contig size distribution (bp). **(B)** Blast hit sequence similarity distribution. **(C)** Blast hit sequence *E*-value distribution. Red line represents the accumulation rate from low value to high whereas green line represents the accumulation ratio from high value to low.

The sequence assembly was performed with the CLC Genome Workbench software (version 4.8). The 104.8 millions of reads were assembled into 150,952 contigs, with an average length of 538 bp, their size distribution is showed in (Figure S1). Out of the assembled contigs, 31,523 with predicted full length CDS were used for further analysis as our reference transcriptome. The size distribution of this subset of sequences is showed in Figure 2A and the list of sequences is available in Additional File 1. Reads of both conditions (drought and control) were individually mapped back to our reference transcriptome and results were used in an RNA-seq assay to do a qualitative analysis (e.g., present/absent of a gene) (Mortazavi et al., 2008). Gene data is available in Additional File 2.

### Functional annotation of reference transcriptome

To obtain a functional annotation, the sequences with predicted full length CDS (*n* = 31,523) were aligned by BLAST against the NCBI NR database and Arabidopsis TAIR10 database using a cut-off *E*-value of 10^−6^. The number of quinoa genes that exhibited homology with the sequences described in the NCBI NR database was 29,334 (93.1%); on the other hand, 2,189 genes (6.9%) did not match the database. These results were different to others results previously reported (Riggins et al., 2010; He et al., 2012; Huang et al., 2012; Zhang et al., 2012; Raney et al., 2014), where the genes without match represented more than 40%. In this case contigs with predicted full length CDS where used but the cited published works included the whole set of contigs. We also cannot rule out that unknown sequences may be novel genes in quinoa, which might be possibly derived from chimerical sequences (assembly's errors), or could be non-conserved regions of proteins. The similarity distribution showed that 41% of the query sequences have a similarity higher than 80%, while 91% of the hits have a similarity higher than 50%, (Figure 2B). The *E*-value distribution of the top hits in the NR database showed that 59.4% of the mapped sequences have strong homology (<1.0E^−63^), whereas 28.6% of the homolog sequences ranged between 1.0E^−7^ and 1.0E^−63^ and only 7% was >1.0E^−3^, which included sequences with no hit (Figure 2C). Those quinoa genes displayed a significant range of identity to sustain that a putative gene function was correlated to a functional gene homology. Indeed, with the very recent availability of a reference quinoa genome (Yasui et al., 2016), out of 31,523 contigs with predicted full CDS, 30,445 mapped to 90,438 genome predicted genes (*e* < 1e^−10^; score > = 500; coverage >= 50%), meaning that our built-in reference transcriptome covers a 40% of the genome transcripts (data not shown).

**Figure 3:**
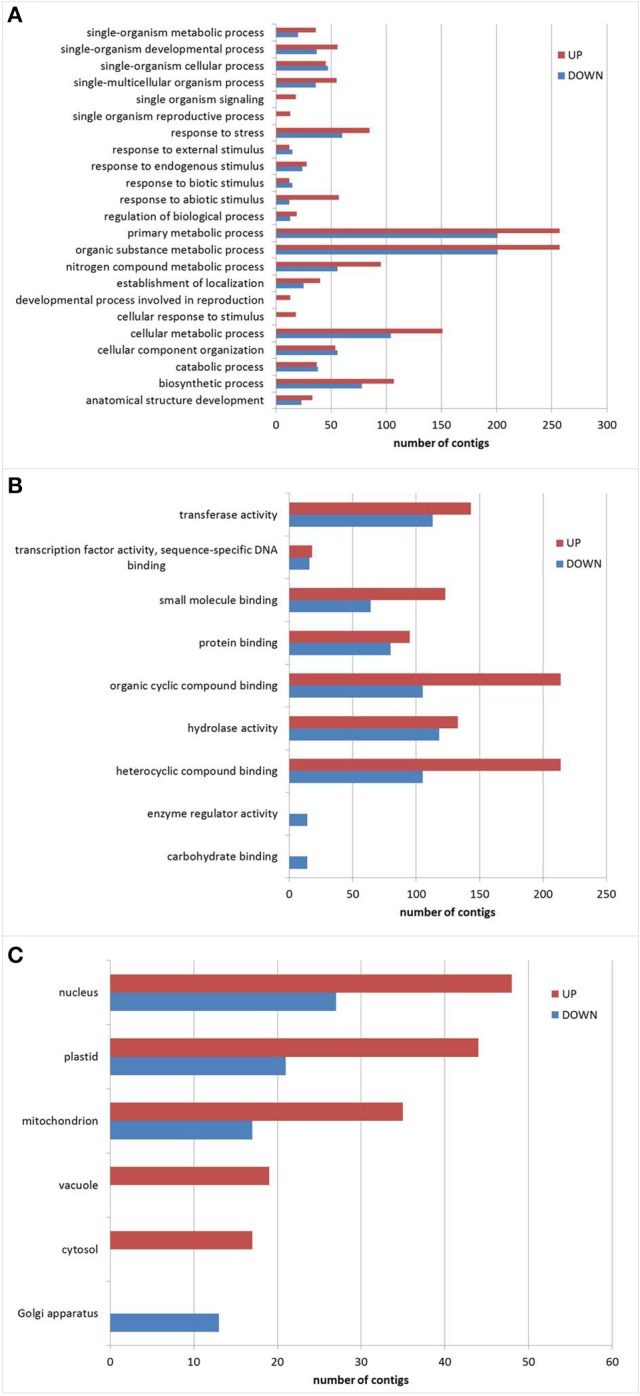
**GO classification of genes with differential expression under drought conditions**. Functional categories **(A)** Biological process; **(B)** Molecular function; **(C)** Cellular component. Red bars: over-represented genes; Blue bars: down-represented genes.

Annotation and determination of gene ontology of transcripts with predicted full length CDS was done by BLAST2GO platform. BLASTX, Interproscan, KEGG and Classification Codes enzymes (EC) were combined to perform a classification with a bigger GO coverage. Additional File 3 contains all the results associated to functional annotation. The results of the GO annotation were used to compare GO categories that changed in response to drought in the expression qualitative subset (2,456 contigs). Figure 3 presents the results for the categories: biological process and molecular function, level 3, and cellular component, level 8. As expected, it was possible to identify a higher number of genes over-represented by drought (85) in the biological process category “response to stress” (Figure 3A) and also 60 genes that were down-represented. In the category “cellular response to stimulus” we found the induction of 18 genes and in the category “response to abiotic stimulus” 57 genes were over-represented and 12 down-represented by drought treatment. Analysis of molecular functions that were affected by drought (Figure 3B), showed a majority of genes related to “organic cyclic compound binding and heterocyclic compound binding,” where 214 of them were over-represented and 105 were down-represented, whereas in “small molecule binding” 123 genes and 64 genes respectively. On the other hand, in the most general category “protein binding” 95 genes were over-represented and 80 genes were down-represented. Also “transferase activity” (with 143 and 113 respectively), “transcription factor activity, sequence-specific DNA binding” (18 and 16), and “hydrolase activity” (133 and 118 genes) in response to drought treatment.

Regarding the “cellular compartment” (Figure 3C), level 8 of Gene Ontology was used to gain insights, and the results showed that the number of genes related to “plastids” and “mitochondrion” that modified their representation was remarkable: 44 genes were over-represented and 21 genes were down-represented for “plastids,” whereas 35 genes and 17 genes were modified for “mitochondrion.” This was followed by the category “nucleus” with 48 genes and 27 genes respectively. Also we found 2 categories that were only present in drought subset: “vacuole” with 19 genes and “cytosol” with 17 genes.

### Gene ontology (GO) enrichment analysis

With each selected gene set we performed a gene ontology enrichment analysis with focus on biological processes terms and found that only GO terms “fruit ripening” and “reproduction” were enriched in drought over-represented genes. Furthermore, “pollen pistil interaction,” “abscission” and “carbohydrate metabolic process” were enriched in drought down-represented genes (Table 2). Indeed, we used 4 X fold change as a cutoff taking in consideration that enrichment analysis did not produce any enriched biological process at 2 X of fold of change.

**Table 2:** **Enriched Biological Processes in drought over-represented gene set or down-represented gene set**.

**Gene set**	**GO code**	**GO Term**	***p*-value**
Drought over-represented genes	GO:0009835	Fruit ripening	1.3E^−02^
	GO:0000003	Reproduction	1.9E^−02^
Drought down-represented genes	GO:0009875	Pollen-pistil interaction	5.5E^−03^
	GO:0009838	Abscission	1.1E^−02^
	GO:0005975	Carbohydrate metabolic process	1.3E^−02^

*Enrichment was determined by Fisher's exact test and the result reduced to most specific terms*.

### RT-qPCR analysis of differentially-expressed genes

**Figure 4:**
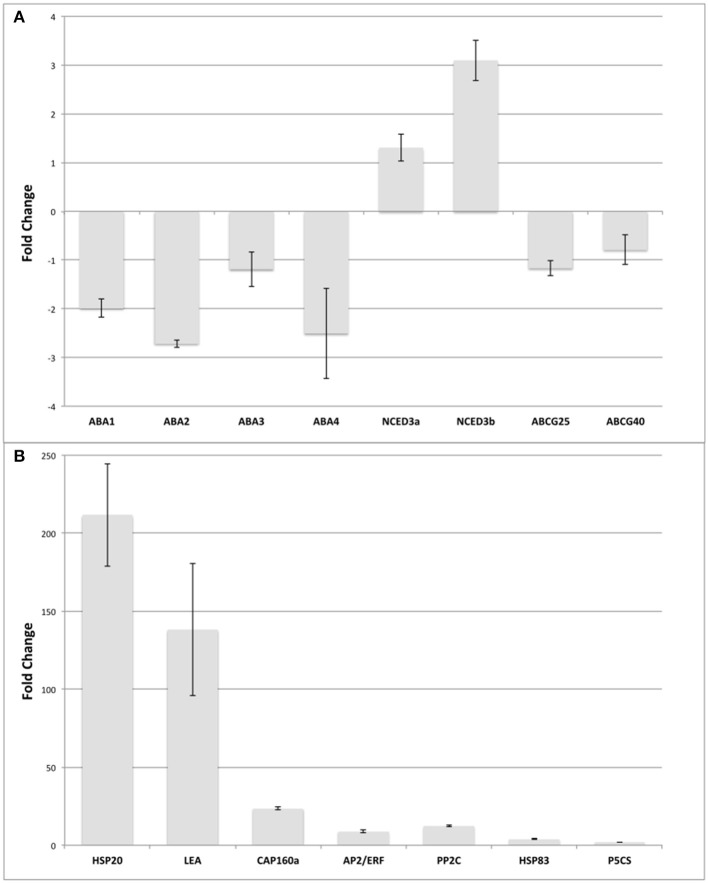
**Gene expression levels measured by qPCR. (A)** ABA transport and biosynthesis genes expression levels. **(B)** Genes that respond to drought. The expression levels are relative to the normalizer gene (Pre-mRNA splicing *PRP18*-interacting factor) that was identified from the *in silico* expression analysis. Bars represent the standard deviation of three replicates.

To confirm both the assembly and the exploratory approach to select target genes, expression analysis by qPCR amplification was performed (target genes and their representation are included in Additional File 1). We also performed Sanger sequencing of all genes used in the qPCR analysis of this work finding a perfect match between the assembly and the sequenced fragment in 90% of the cases (see Additional Files 4, 5). On the other hand, when mapping the predicted contigs with full CDS against the Quinoa genome (Yasui et al., 2016) we found that 90% had a structure according to the genome (*e* < 1e^−10^; score > = 500; coverage > = 50%). The gene selection was made based on two criteria. First, genes that were induced under drought conditions in other plant models were identified through literature mining. In addition, we attempted to reconstruct a canonical pathway associated with drought stress, the Abscisic Acid (ABA) pathway. The ABA biosynthesis pathway was used as a reference, corresponding to the described by Seo and Koshiba (2011). This pathway consists of 5 genes, three of which are located in the plastids: *ABA1, ABA4* and *NCED3* and two in the cytosol: *ABA2* and *ABA3*. In addition to ABA biosynthesis pathway genes, two important genes involved in ABA transport were included, *ABCG25* and *ABCG40*. Others genes involved in stress response were selected based in the changes of representation reads detected by RNA-Seq in quinoa: *CqHSP20* (putative chaperones hsp20-protein superfamily), *CqCAP160* (cold acclimation protein 160), *CqLEA* (late embryogenesis abundant protein family protein), *CqAP2/ERF* (integrase-type DNA-binding protein superfamily), *CqPP2C* (protein phosphatase protein family 2c), *CqHSP83* (chaperone protein, protein family HTPG), and *CqP5CS* (delta 1-pyrroline-5-carboxylate synthase 2). To address the *RNA-Seq* changes, expression analysis by qPCR of selected 15 unigenes were performed. The qPCR analysis showed a similar pattern to the *in silic*o analysis (Figure 4). We determined that *CqNCED3a* and *CqNCDE3b* were the only genes involved in the ABA biosynthesis pathway that were up-regulated by drought in quinoa. For *CqABA1 and CqABA3*, we determined slight down-regulation by drought (≤2 Fold Change), whereas *CqABA2*, and *CqABA4* were down-regulated (>2 Fold Change). Similar slight down-regulation were determined for *CqABCG25* and *CqABCG40*, involved in ABA transport (Figure 4A). In addition, the genes *CqHSP20, CqLEA, CqCAP160, CqAP2/ERF, CqPP2C, CqHSP83*, and *CqP5CS* were up-regulated at variable magnitudes in response to drought conditions as Figure 4B shown, which is in agreement with previous reports in different plant models (Kaye et al., 1998; Wang et al., 2004; Ali-Benali et al., 2005; Nakano et al., 2006; Swindell et al., 2007; Umezawa et al., 2009; Sharma and Verslues, 2010; Merewitz et al., 2011; Candat et al., 2014). Interestingly, *CqHSP20* and *CqLEA* experienced a shift over 140-fold expression level, whereas other group of genes (*CqCAP160, CqAP2/ERF, CqPP2C, CqHSP83*, and *CqP5CS*) the expression level change was between 24 and 2-fold respectively.

## Discussion

Quinoa or quinua (*Chenopodium quinoa* Willd.) is vastly distributed throughout the Andean region covering a range of 12,000 km from Colombia to Chile, also defining five ecotypes: inter- Andean valleys, Highlands, Yungas, Salares (salt flats), and coastal/lowlands. Due to the geographical diversity of our country, several genotypes can be found in the only two ecotypes present: Salares, which is distributed in the Tarapacá and Antofagasta regions (18–25°S), with elevations over 3,000 m high and precipitation fluctuating between 100 and 200 mm per year falling during the southern hemisphere summer, whereas coastal/lowlands, the latter being the only temperate latitude ecotype is distributed in central Chile and more southern latitudes (43°S) (O'Higgins to Lakes' regions) and are rain-fed with variable altitudes between sea level and 1,000 m height. A remarkable difference is that compared to the extremely dry conditions where the “Salares” quinoa is grown in northern Chile, rainfall in the central and southern zones of Chile occurs during the southern hemisphere winter (June–August), with rainfall fluctuations between 500 and 2,000 mm per year. This rainfall increases steadily across 34–40°S (Martínez et al., 2015). Among this diversity it is possible to distinguish two groups through genetic distance, most probably due to the lack of seed exchanges: “coastal/lowlands” and “Salares” (Fuentes et al., 2008; Martínez et al., 2015). Therefore, it was necessary to perform a selection among the Chilean genotypes that could exhibit distinctive tolerance to drought. Three representative Chilean genotypes were assessed to select the most drought tolerant: R49 (Salares), PRJ (coastal/lowlands), and BO78 (coastal/lowlands). These genotypes represent three bio geographical areas or growing zones (north, central and south) previously described (Fuentes et al., 2008, 2012). After the analysis of relative water content, electrolytic leakage and maximum efficiency of photosystem II, we determined that the R49 genotype was the one with the best performance on physiological parameters selected and the highest tolerance to drought, considering that decline in photosynthetic parameters occurred concurrently with the appearance of physical symptoms of drought stress, including water loss and membrane stability among others.

The quinoa R49 transcriptome was sequenced by the Illumina pair-ends method, using total RNA extracted from quinoa plants under stress and from total RNA extracted out of control plants. We obtained 104.8 millions of reads, which were assembled into 150,952 contigs with an average size of 538 bp, where 18,124 contigs had a higher or equal length of 1,000 bp and 132,826 contigs were less than 1,000 bp in length. The high percentage of contigs less than 1 Kb is expected on this type of approach (*de novo* assembly) were there were no reference transcriptome to date of experiments and also due to the polyploidy of this specie (Crawford et al., 2010; Mizrachi et al., 2010; Zhang et al., 2012). Taking in consideration that a high number of these contigs could be fragments of genes, we decided to select those contigs that has been predicted as full length CDS (*n* = 31,523 contigs). To determine the identity of those predicted full length CDS quinoa contigs, BLAST, Interproscan, KEGG and EC tools were used. The results indicated that 6.9% of the analyzed genes had no identity with any other gene in the public databases used in this study. This is a small number of unknown genes compared to other similar reports but the difference may lay in the fact that we used only a subset of contigs with predicted full length CDS to ensure more confidence avoiding assembly problems related to polyploidy. Among the contigs with known identity, we observed a small percentage having a low homology; therefore we assumed that the function of the genes was indeed the one predicted for the orthologous gene.

We indeed found six processes that could be reduced to two main biological processes: “fruit ripening” and “reproduction” under a 4 X fold change criterion and FDR *p* < 0.05, 2,456 differentially represented genes were identified, from which 1,579 were over-represented and 877 were down-represented under drought conditions. By analyzing the genes identity, we found 76 genes with unknown identity that were down-represented under drought, equivalent to 9% of the total of genes that were down represented, whereas 306 genes with unknown identity were over-represented under this stress, equivalent to 19% of the total of genes over-represented in drought. When we searched for the number of drought-induced genes in other models such Arabidopsis (Seki et al., 2002), the number of genes with known identity was one fifth to the ones induced in quinoa under the same stress (277 vs. 1,273 respectively). By taking a sample of the genes population, it was expected that the percentage of genes without identity would be conserved from what is observed in the entire population, in our case around 6.9%. We finally determined that among drought over-represented transcripts, genes with unknown identity were 19%, well above the expected 6.9%. These results allow us hypothesized that the drought response in quinoa might present several unknown paths. Recently, Raney et al. (2014) published the transcriptome sequencing of two quinoa genotypes in response to drought. These authors obtained a lower number of genes in comparison to those reported in our work, i.e., 462 differentially expressed contigs identified upon water stress treatments, 251 which presented sequences that could be annotated with a functional gene ontology and assigned to a GO category, despite the use of a combination of methodological approaches, including 454 and ESTs sequencing, and different genotypes analyzed (Raney et al., 2014). Indeed, these authors identified a group of 27 differentially expressed genes that can be classified as having regulatory functions, including one whose inferred polypeptide product showed high amino acid sequence homology to Naringenin, 2-oxogluturate 3-dioxygenase, which is intermediate in the biosynthesis of flavonoids in plants (Raney et al., 2014). It is interesting that the two GO terms enriched on the drought over-represented genes, the processes of “fruit ripening” and “reproduction,” has been associated to drought stress in grapevine, cereals and other plants on studies related to climate change (Castellarin et al., 2007; Barnabas et al., 2008; Chaves et al., 2010; Fleury et al., 2010) where they have been related to an escape strategy before the onset of severe drought stress. This strategy involves several biological processes, most of them directed to dehydration avoidance like minimized water loss (e.g., caused by stomatal closure, trichomes, reduced leaf area, senescence of older leaves, etc.) or maximized water uptake (e.g., by increased root growth). These changes must be equilibrated energetically and we found that, under drought condition quinoa also displayed reduction in pollen-pistil interactions, possibly related to CLE genes in response to environmental stimuli such as heat stress (Wang et al., 2016), reduction in abscission processes and reduction in carbohydrate metabolic processes suggesting different ways of metabolic plasticity under the drought condition.

The results obtained from our *in silico* analysis served as reference to further identify target genes for *in vivo* experiments, where the expression patterns of 15 genes were assessed by qPCR, which were chosen for canonical responses to drought such as ABA biosynthesis, since ABA regulation seemed to be one of the mechanisms utilized by quinoa when facing drought induced decrease of turgor of stomata guard cells (Jacobsen et al., 2009), and another group of genes directly induced in response to drought. We could determine that *CqABA1, CqABA2*, and *CqABA4* from the ABA biosynthesis pathway were repressed over two times. One hypothesis to explain why these two genes were repressed might be that in quinoa a family of corresponding genes might be present as tetraploid species, and the designed primers did not differentiate between the members of the family. A previous study concluded that ABA apparently plays a minor role under drought conditions in quinoa, and the authors suggested that quinoa might produce other antitranspirants compounds than ABA in the xylem sap (Jacobsen et al., 2009). Also, hormonal stress signals may exist and may play an important role in quinoa, suggesting that both cytokinin and ethylene reactions should be further dissected in quinoa.

The relative abundance of studied target genes was highly variable in response to stressful conditions. There were genes that had very low expression levels, as ABA-pathway transcripts described above. In the other hand, the highest up-regulation (over 200-fold) was exhibited by *CqHSP20*, might indicates that drought treatment affected the thermal regulation via water shortage, since *Hsp20* genes have been induced to a larger extent in tomato plants under various abiotic stresses, including heat, salt, and drought treatments (Yu et al., 2016), which may suggest that plants were inducing ABA-independent responses as well. Small HSPs (sHSPs) are involved in folding and assembling protein, keeping protein stabilization, activating protein, and degrading protein in many normal cellular processes and under stress conditions. Indeed, most of the HSPs (HSP18.1, HSP18.3, HSP20, HSP21.7, HSP25.3, HSP26.26, and HSP30) were significantly up-regulated during the heat treatment in spinach (*Spinacia oleracea* L.) leaves under heat stress (Yan et al., 2016). In the case of *CqLEA* gene that was induced 140-fold in treated plants, consistent with the fact that the ABA-responsive element was first described for a group 2 LEA gene from rice, there are genes for this group of proteins whose expression in response to stress is mediated by ABA. Moreover, there are examples of dual regulation; that is, their response to stress is mediated by more than one pathway, one of which may be ABA dependent (Welling et al., 2004; Battaglia et al., 2008). A different case was *CqAP2/ERF*, whose up-regulation might be pointing to a pivotal role in coordination of regulatory networks underlying abiotic stress tolerance (Golldack et al., 2014). Furthermore, functional analysis of berry transcriptomic responses to higher temperatures revealed the induction of heat shock protein (HSP) chaperones coincident with up-regulation of ERF subfamily transcription factors and increased ABA levels, suggesting their participation in the maintenance of the acclimation response (Carbonell-Bejerano et al., 2013).

Differentially abundant genes both over-represented and down-represented in drought treatment were compared among them using Blast2GO. The categories that shown over-represented genes that are commonly associated to drought were “response to stress” as expected with 85 genes and “response to abiotic stimulus” with 57 genes. Among the biological processes that were found in both subsets but over-represented in drought we highlight: “biosynthetic process,” “single-organism metabolic process,” “nitrogen compound metabolic process,” “establishment of localization,” “single-multicellular organism process” and “single-organism developmental process.” Also there were four processes only detectable in the over-represented subset: “cellular response to stimulus” (18 genes), “single organism signaling” (18 genes), “developmental process involved in reproduction” (13 genes) and “single organism reproductive process” (13 genes). In general, these results coincide with reports for *Thellungiella*, wheat, poplar, rice, cassava, *Arabidopsis* and chickpea (Wong et al., 2005; Mochida et al., 2006; Street et al., 2006; Gorantla et al., 2007; Lokko et al., 2007; Huang et al., 2008; Varshney et al., 2009). These results indicated that in quinoa most responses to drought are conserved, as for most species where this abiotic stress has been studied. The difference in tolerance levels might rely in the population of genes without identity. When looking at categories of genes that were regulated with stress, we highlighted the plasma membrane related genes, because these variations can be linked to cell turgor maintenance (Razzaghi et al., 2015) or membrane stability observed by measuring electrolyte leakage. Indeed, we found an up-representation of transporter activity genes such as *ABC, ERD6-like, MATE, SWEET-like* among others, in parallel with down-representation of cell wall modifying genes. These variations can be attributed to the ability to withstand stress levels to which the plant was submitted, remobilization of assimilates and might be linked to ROS (reactive oxygen species) detoxification, since they cause severe cellular damage by peroxidation and de-esterification of membrane-lipids (Golldack et al., 2014; Raney et al., 2014).

## Conclusions

This work has determined that quinoa transcriptome from a tolerant genotype R49 has exhibited a 15% of genes (382 contigs) that not presented homology to the published databases from 2,456 identified differentially represented transcripts under drought conditions. The over-represented genes were higher (1,579) than down-represented genes (877) by drought treatment, and 19% of over-represented genes (306 contigs) were unknown. In Arabidopsis, approximately 40% of enzyme- and transporter-encoding genes have credible functional annotations, and this number is even lower in non-model plants. The slight up-regulation of ABA genes in response to drought stress in quinoa might indicate that ABA-independent mechanisms are committed to coordinate responses to acclimate to hydric deficit. Also functional characterization of unknown genes remains a challenge, but various databases and homologs cross-kingdom comparative genomics could be mined to provide clues (Niehaus et al., 2015). Therefore, integrative efforts are still necessary to unravel how this resilient species is able to withstand in adverse environments where others species fail to succeed. Indeed, the information represents a very useful tool for selecting drought tolerant parentals or lines with active tolerance mechanisms for breeding purposes, being useful to explore the differentially expressed gene space and valuable for loci identification in ongoing quinoa breeding efforts.

## Author contributions

AM performed drought treatments, prepared RNA samples and interpreted the results. JM performed assembling, expression analysis and interpreted the results. AM, HS, and AZ designed the experiment and provided guidance of the study. AZ and HS provided assistance in the data analysis, management data interpretation and wrote the manuscript. All authors have read and approved the final manuscript.

### Conflict of interest statement

The authors declare that the research was conducted in the absence of any commercial or financial relationships that could be construed as a potential conflict of interest.

## Supplementary material

Figure S1 **Distribution of contigs size obtained from the genome reference (hybrid assembly)**.

Table S1 **Soil water potential for quinoa plants ecotype R49**.

Table S2 **qPCR primers list for selected genes in this study**.

Additional File 1 **FASTA file with predicted full length CDS contigs used for further analysis as our reference transcriptome**.

Additional File 2 **Excel spreadsheet with differentially expressed genes (DEG analysis) when compared reads from drought and control conditions**. Proportions fold of change of values minus infinite and plus infinite was set to −1,000,000 and 1,000,000 respectively.

Additional File 3 **Excel spreadsheet with the annotation and gene ontology of transcripts with predicted full length CDS**.

Additional File 4 **Excel spreadsheet with the validation of qPCR amplicons by BLAST alignment against quinoa transcriptome**. Amplicons were sequenced using Sanger technology.

Additional File 5 **Excel spreadsheet with the structure validation of selected quinoa genes by BLAST alignment against the genome predicted transcripts (Yasui et al., 2016)**.

## Availability of data and materials

The nucleotide sequences of raw reads from this study were submitted to NCBI's Sequence Read Archive through the BioProject ID: PRJNA305752. Contigs with predicted full length CDS are available in Additional File 3.

## Abbreviations

RWC: Relative Water Content
EL: Electrolyte Leakage
qPCR: Quantitative Real-Time PCR
ABA: Abscisic acid
BP: Biological process
FC: Fold-change
GO: Gene ontology.
