# Supplementary figures and images for: Transcriptional Responses of Chilean Quinoa (Chenopodium quinoa Willd.) Under Water Deficit Conditions Uncovers ABA-Independent Expression Patterns
Source: Front Plant Sci. 2017 Mar 8;8:216. doi: 10.3389/fpls.2017.00216 (PMC5340777; doi:10.3389/fpls.2017.00216)

**Figure S1: Distribution of contigs size obtained from the genome reference (hybrid assembly).**

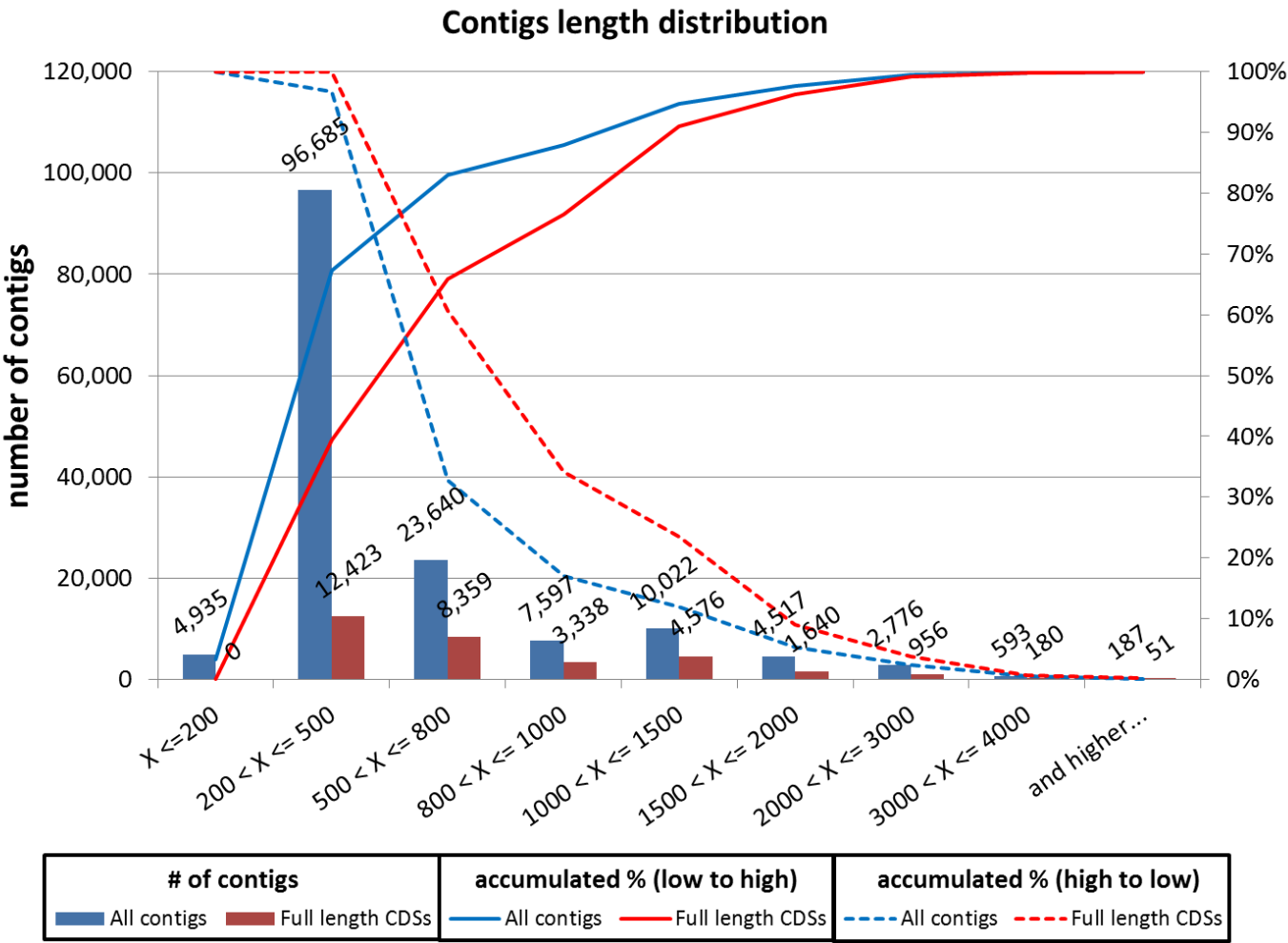

Supplement: Figure S1 — Distribution of contigs size obtained from the genome reference (hybrid assembly). [file Image1.pdf]
