# Supplementary material for: Transcriptional Responses of Chilean Quinoa (Chenopodium quinoa Willd.) Under Water Deficit Conditions Uncovers ABA-Independent Expression Patterns
Source: Front Plant Sci. 2017 Mar 8;8:216. doi: 10.3389/fpls.2017.00216 (PMC5340777; doi:10.3389/fpls.2017.00216)
Supplement: Table S1 — Soil water potential for quinoa plants ecotype R49. [file Table1.docx]

**Table S1: Soil water potential for quinoa plants ecotype R49**

| Days after stress | Control (C) | | Drought (D) | |
| --- | --- | --- | --- | --- |
|  | Ψ soil (MPa) Expected | Ψ soil (MPa) Measured | Ψ soil (MPa) Expected | Ψ soil (MPa) Measured |
| 2^a^ | -0.24 | -0.6 | -1.16 | -1.3 |
| 3 | -0.30 | -0.6 | -1.34 | -1.6 |
| 4^b^ | -0.28 | -0.5 | -1.43 | -1.3 |
| 5 | -0.79 | -0.9 | -1.57 | -1.5 |
| 6 | -0.31 | -0.6 | -2.92 | -9.6***** |
| 7 | -0.16 | -0.5 | -1.64 | -1.5 |
| 8^c^ | -0.25 | -0.6 | -2.66 | -2.7 |
| 9 | -0.29 | -0.5 | -2.62 | -2.7 |

^a^: correspond to C1 and D1 for sampling; ^b^: correspond to C2 and D2 for sampling; ^c^: correspond to C3 and D3 for sampling.
