# Supplementary material for: Transcriptional Responses of Chilean Quinoa (Chenopodium quinoa Willd.) Under Water Deficit Conditions Uncovers ABA-Independent Expression Patterns
Source: Front Plant Sci. 2017 Mar 8;8:216. doi: 10.3389/fpls.2017.00216 (PMC5340777; doi:10.3389/fpls.2017.00216)
Supplement: Table S2 — qPCR primers list for selected genes in this study. [file Table2.docx]

**Table S2.** qPCR primers list for selected genes in this study

| **Gene** | **Primers for qPCR** |
| --- | --- |
| *ABA1* | F5’-CCACTTACCAACAATACTCTCATC-3’  R5’-TCTGCCCACTAAATGTTGACTT-3’ |
| *ABA2* | F5´-CTTACTCTGCTCCAAACTCC-3’  R5’-AACGCACAATGCTCTCTC-3’ |
| *ABA3* | F5´-GAACAAGCAGTTGAGAGGTG-3’  R5´-TAGCCAAGGCAGTTTGAG-3’ |
| *ABA4* | F5’-CCTTCTGTACGCTTATCTTCTGT-3’  R5’-GAATCCATGCTGATGCTAATGTC-3’ |
| *NCED3b* | F5´-GCAAACATCCCTCTGGTA-3’  R5´-CTTATGGGTTCCACGGTA-3’ |
| *NCED3a* | F5’-CGATGAGGTGGTGGTGAT-3’  R5’-CGCCCGAGTTTATTTCTG-3’ |
| *ABCG25* | F5’-GTCCGAGAAACACTCCTCTAC-3’  R 5’-CTCCCATAACAGACTCAACG-3’ |
| *ABCG40* | F5’-ATGGACTGATTGACGGATTGAG-3’  R5’-GGTAGGAGATGAGATGATTAGAGG-3’ |
| *HSP20* | F 5´-CGGGGCTAAAGAAGGAAG-3’  R 5´-TGCCACTTATCGGTCTCC-3’ |
| *LEA* | F5´-CTTGGCTTCCTCTTTCTTCT-3’  R5´-TGAGACTGCTGATGCTACTG-3’ |
| *CAP160a* | F5´-CGAGTAACGGAGTCAGAAGA-3’  R5´-CCAGAAAGTAGCAGCATCTC-3’ |
| *AP2/ERF* | F5´-TGACACACCTCTATCCACAG-3’  R5´-AGACTGAGAAGCCGATGTAG-3’ |
| *PP2C* | F5´-AATGAGGAAGTGGGAGGT-3’  R5´-CAAAGGAACAGCAACACC-3’ |
| *HSP83* | F5´-AACACCCTCCTGACATACAG-3’  R5´-GACCCTCTTCACTGGAAAC-3’ |
| *P5CS* | F5´-CATTGGGTGTTCTCCTGA-3’  R5´-TCTCTTTGCCTCCTTTCC-3’ |
